# HPV Vaccine Uptake and Cervical Cancer Trends in Panama: A Reference Point for Future Impact Studies

**DOI:** 10.3390/vaccines13111173

**Published:** 2025-11-19

**Authors:** Arlene Calvo, Sabrina Hall, Verónica B. Melgar Cossich, Jonathan Andreadakis, Humberto López Castillo, Dalys Pinto, Itzel de Hewitt

**Affiliations:** 1 College of Public Health, University of South Florida, Tampa, FL 33612, USA; shall5751@gmail.com (S.H.); veronicamelgar09@gmail.com (V.B.M.C.); jandreadakis@usf.edu (J.A.); 2 Health Services Advisory Group, Phoenix, AZ 85016, USA; 3 Department of Health Sciences, College of Health Professions and Sciences, Academic Health Sciences Center, University of Central Florida, Orlando, FL32816, USA; dr.hlc@ucf.edu; 4 Department of Population Health Sciences, College of Medicine, Academic Health Sciences Center, University of Central Florida, Orlando, FL 32827, USA; 5 Essential Program on Immunization (EPI) of Panama, Ministry of Health, Panama City 0816-02593, Panama; dalysibethp@yahoo.com (D.P.); ihslocum@minsa.gob.pa (I.d.H.)

**Keywords:** HPV vaccine, vaccine uptake, cervical cancer, Pap tests, Panama

## Abstract

**Background**: Cervical cancer (CC) continues to be an important public health concern in Latin America, where it is the second cause of cancer-related deaths among women. With its strong culture of vaccination, Panama was the first country to implement the HPV vaccine as part of its Essential Program on Immunization (EPI). Recently, the government implemented the 90:70:90 PAHO/WHO strategy to reach milestones toward CC elimination. **Objective**: This analysis triangulates and assesses national data on HPV vaccination coverage, screening practices, and cervical cancer incidence and mortality in Panama, to understand historical tendencies to date and establish a comprehensive foundation for future impact evaluations and research studies. The analysis aims to identify trends and gaps in prevention efforts and to serve as a reference point for future research on HPV-associated cancers. **Methods**: Population-based, descriptive, observational, ecological study where four, aggregate, de-identified data sources by various curators in Panama were match-merged by year, sex, and administrative division. Reported outcomes include HPV vaccine coverage, CC incidence and mortality rates, screening Pap tests, and CC behavior at diagnosis (in situ vs. invasive). **Results**: Panama has high HPV vaccine uptake (≥85% most years) in spite of low Pap test coverage (~10%). A decreasing trend in CC incidence has been observed continuously since the 1990s, counterintuitively to significantly increasing CC mortality rates, with most cases diagnosed as invasive and among younger women (30–69 years old). **Conclusions**: This report provides a comprehensive foundation for understanding trends in HPV vaccination coverage, cervical cancer incidence and mortality, and screening practices in Panama. While high vaccine uptake and declining incidence trends are encouraging, persistent low screening rates and elevated mortality—particularly at invasive stages among younger women—highlight critical gaps in prevention efforts. The need for integrated strategies that strengthen data systems, improve early detection, and address structural and sociocultural barriers are discussed, framed within Panama’s progress toward achieving the 90:70:90 targets. Future studies should focus on understanding non-medical influences on health and further vaccine impact with patient-level data, and other forms of HPV-related cancers in immunosuppressed populations. Public strategies would benefit from the implementation of real-life data and efficient data management, consolidation systems, systematic health promotion interventions, and an increase in resource allocation for women at the highest risk.

## 1. Introduction

Cervical cancer (CC) continues to pose an important threat to the health of Latin American and Caribbean (LAC) women, especially in Panama, where CC is the second leading cause of cancer-related death, with breast cancer being the first [1]. CC is generally a slow-growing cancer that develops in the cells lining the endocervical canal [2,3]. The disease typically begins with a persistent high-risk human papillomavirus (HPV) infection, which can lead to the development of squamous intraepithelial lesions [4,5]. Globally, CC is the fourth most prevalent cancer to impact women, with an estimated 660,000 women affected, resulting in approximately 350,000 deaths in 2022 [4,6].

Persistent HPV infections contribute significantly to the etiology of CC, accounting for 99.7% of CC diagnoses [5,6,7,8,9,10,11]. Over 200 HPV strains are currently identified and categorized into two main types of risk categories for HPV infections: non-oncogenic and oncogenic [5,6,7,8,9,10,11]. Non-oncogenic HPV infections cause oral, genital, or anal warts [6], while HPV types 16, 18, 31, 33, 35, 39, 45, 51, 52, 56, 58, and 59 are high-risk oncogenic strains causing persistent infection that alter cervical cells, significantly increasing the risk of malignant lesions, most commonly by types 16 and 18 [9,10]. The timeline between HPV infection and the onset of the malignancy depends on the host’s immune response; for this reason, HPV infection is also studied in other forms of cancers and in adults living with HIV [11,12,13]. In general, preinvasive lesions may appear as early as two years after infection, while the development of invasive cancer lasts longer than ten years [5,6,7,8,9,10]. The high-risk oncogenic types are of special interest in prevention through immunization [4,10].

Women in low- and middle-income countries (LMICs) are proportionally more impacted by CC than women in high-income countries, representing 94% of CC fatalities [3,7,9,10]. LAC is a region that combines LMICs, with incidence and prevalence rates of CC exceeding the global average [14,15,16,17,18,19,20]. The CC mortality risk in LAC is currently three times higher than in North America [16,17,19]. Over the past three decades, extensive regional and national efforts in LACs have attempted to reduce the population’s CC risk [15,20]. In 2008, programs were identified across LAC to implement HPV vaccination and raise CC awareness [18,21,22,23]. At the time, CC was the leading cause of cancer-related deaths among women in most LAC countries, including Panama [18,19,20,21,23,24]. Currently, in Panama, the average annual incidence rate of CC is 16.1 per 100,000 women, more than twice of the United States (7.7 per 100,000 women) [1,19,20,21,24].

Panama is a country located in Central America, with a population of approximately 4.5 million [25]. The country is divided into ten provinces and six indigenous comarcas, which in turn are subdivided into districts and corregimientos (smaller geopolitical administrative regions, akin to a county). Most of the population concentrates in the larger metropolitan urban (Metropolitan Panama and Colón) and peri-urban areas (Panamá Oeste, Panamá Este, Panamá Norte, San Miguelito), while the rural areas are less densely populated (Chiriquí, Herrera, Los Santos, Coclé, Darién) [25].

Attempting to lessen the high incidence and deaths due to CC, Panama introduced the HPV vaccine into the Ministry of Health’s (MoH) Essential Program on Immunizations (EPI) in 2008, becoming the first country to implement the HPV vaccine [21,22,26]. The initial implementation was a national, compulsory, three-dose, bivalent vaccine series administered to 10-year-old girls through the MoH School Health Program [26]. All EPI vaccines are administered with no out-of-pocket cost in schools or MoH community health clinics, and for a fee in private clinics [26]. In 2014, the EPI schedule continued with girls and key populations, and switched to a two-dose, bivalent vaccine scheme and subsequently, in 2015, to the two-dose, quadrivalent vaccine. In 2016, ten-year-old boys were included as part of the HPV vaccine program, with the same two-dose, quadrivalent vaccine scheme [26]. The male component is an important aspect of HPV transmission, as originally studied in Panama, supporting the concept of gender-neutral HPV vaccination and the importance of herd protection [27,28,29]. Panama’s EPI included the nine-valent vaccine for boys and girls as part of their schedule, during the 2025 Vaccination Week in the Americas (VWA) [30]. Furthermore, Panama was the first country in Spanish-speaking America to introduce the nine-valent vaccine [30]. Also in 2025, the MoH of Panama, with support from the Pan-American Health Organization/World Health Organization (PAHO/WHO), locally launched the global 90:70:90 Strategy to provide adequate milestones to eliminate cervical cancer by the year 2030 [31]. The plan includes improvements in HPV screening, strengthening primary care, expanding cancer services, and providing technical and financial support for vaccines, supplies, and data collection with the goal of achieving 90% vaccination coverage, 70% cervical cancer screening at least twice in a lifetime, and 90% treatment of pre-invasive lesions and invasive cancers [30,31]. The present report synthesizes data on HPV vaccination coverage in girls and boys, and CC screening, incidence, and mortality in Panama to generate a baseline, descriptive report in tandem with the introduction of the HPV vaccine in the EPI. The objective of this analysis is to triangulate and assess national data on HPV vaccination coverage, screening practices, cervical cancer incidence, and mortality in Panama, to understand the current tendencies and establish a comprehensive foundation for future impact evaluations and research studies. The analysis aims to identify trends and gaps in prevention efforts and to serve as a reference point for future research related to HPV-associated cancers.

## 2. Materials and Methods

### 2.1. Study Design

This is a population-based, descriptive, observational, ecological study. To achieve the study aims, we gathered multiple, aggregate, de-identified data sources, described next.

### 2.2. Data Sources

The team, assisted by local partners, retrieved four, primary, aggregate, population-level datasets: (1) Panama’s HPV vaccination coverage dataset, curated by Panama’s EPI and routinely collected through immunization cards registered by the EPI nurses and reports from immunization campaigns, both of which are regularly transcribed into a centralized, national EPI database [26]; (2) Panama’s Pap test screening dataset, curated by the MoH’s Sexual and Reproductive Health (SRH) Program [32,33]; (3) the CC case counts and incidence and mortality rates estimates, curated by the National Cancer Registry [34], which receives all national cancer data from 33 sources that include public and private hospitals and clinics and serves as a central repository for cancer information [35]; and (4) Panama’s population estimates by year, sex, age groups, and provinces, curated by the National Institute for Statistics and Census (INEC) of the General Comptroller of the Republic of Panama [25,36]. Links to these publicly available datasets are provided in the Data Availability Statement section.

### 2.3. Data Quality Assurance

Routine formal quality assessments are not reported by curators of the primary datasets and are beyond the scope of this report. However, as an initial step towards data quality assurance, the authors relied on descriptive checks aligned with WHO’s Data Quality Review framework [37] and International Agency for Research on Cancer (IARC) [38] guidelines for immunization programs and population-based cancer registries, focusing on the structure, completeness, coverage, consistency, plausibility, external concordance, and timeliness of the datasets. These guidelines enhance transparency regarding publicly available dataset quality—especially in terms of reliability and accuracy—albeit advanced validation metrics (e.g., morphologically verified or death-certificate-only cases) were not available for all data sources every year.

In terms of completeness, all ten provinces and six indigenous comarcas were represented in the immunization and screening datasets. However, historical gaps were noted in cancer registry data. These gaps were flagged and treated descriptively without imputation. Regarding coverage and plausibility, administrative coverage occasionally exceeded 100%, a recognized phenomenon in immunization programs in Panama due to mass campaigns, duplicate records, non-registered floating migrant populations (e.g., >520,000 in 2023), and denominator inaccuracies. These anomalies were examined for plausibility and are discussed in this manuscript in the context of Panama’s vaccination practices and census limitations, as has been reported in other vaccine studies [39]. Temporal consistency was evaluated by examining year-to-year trends for vaccination, screening, and cancer incidence. Joinpoint regression was applied to cancer incidence and mortality rates to identify significant changes in trends over time. Screening and vaccination data were reviewed for abrupt fluctuations that might indicate reporting inconsistencies or programmatic disruptions (e.g., during the COVID-19 pandemic). In terms of external concordance, where feasible, immunization and cancer registry figures were compared with international benchmarks, including estimates from the WHO/UNICEF Joint Reporting Form on Immunizations (JRF) [40] and PAHO regional reports [41] on immunization data and statistics.

These comparisons provided a relative check on the alignment of Panama’s reported data with global patterns, though formal concordance analysis was limited by data availability. Last, we documented the reporting processes for each data source to assess timeliness and reliability. As mentioned, immunization data are collected through nurse-registered cards for clinic or community tallies, transcribed into a centralized EPI database, and reported annually to PAHO/WHO. While formal lag analysis was not performed, these processes suggest structured reporting cycles, though delays in Pap test result entry and registry updates are noted and discussed as potential limitations.

### 2.4. Study Variables

The study variables were those that allowed the estimation of the study outcomes. To the extent available, these population-level variables were recorded by year, sex, age, vaccine dose, and geographical location. Variables included: counts and types of administered HPV vaccine doses (first, second, and third, where applicable), counts of Pap tests conducted, counts of new cases of CC along with their respective tumoral behavior at the moment of diagnosis (in situ vs. invasive), number of deaths attributed to CC, and population estimates that serve as denominators for certain study outcomes, described next.

### 2.5. Study Outcomes and Measures

From the population-level variables, the following outcomes are reported by year, sex, and province or comarca to the best extent that the data allow:

- Vaccine uptake. In absolute numbers, measured by the number of first, second, and third doses (when applicable) of HPV administered by sex and year.
- Vaccine coverage. The EPI estimates the vaccine coverage by dividing the number of doses in the vaccination age group and province or comarca by the INEC’s population estimates for the same year, sex, and age group and province or comarca.
- CC screening. The SRH Department at the MoH provides aggregate data on Pap tests conducted, which are divided by INEC’s population estimates for women ≥18 years old.
- CC cumulative incidence rate. Estimated by dividing the counts of CC cases by the year’s population for each demographic subgroup.
- CC mortality rate. The mortality rate by CC was estimated by dividing the total number of deaths attributed to CC each year by the mid-year population estimate of women ≥18 years old. The counts of new CC cases are also described by age groups and by years of diagnosis.
- CC tumoral behavior at diagnosis. Tumoral behavior at the time of diagnosis of CC was classified as in situ or invasive and reported as counts per year. Cancer stages are unidentified in the current data.

### 2.6. Statistical Analyses

All datasets were shared and downloaded from their respective curating source and organized and merged by year, sex, and province or comarca, as applicable. Descriptive statistics were reported for counts as frequencies and percentages. Trends and comparative analyses were applied to assess changes in CC incidence before and after implementing the HPV vaccination program. Data were assessed to estimate the program’s course over the past 17 years, providing an inspection of the HPV vaccination program. HPV vaccine uptake and CC screening through Pap tests were analyzed, using the Chi-square test of independence by year and geographic location, time series plots, and Joinpoint analysis of annual percentage changes (APCs). CC cumulative incidence rates between 1990 and 2023 were collected and graphed to illustrate the changes in incidence rates per 100,000 over time in Panama.

We analyzed annual HPV dose 1 uptake by sex using a time-series regression on the logit scale. Let $r_{t,s}$ denote the uptake proportion in calendar year t for sex s (girls =0, boys =1). We modeled:

$$\mathrm{logit}\left(r_{t,s}\right)=\beta_{0}+\beta_{1}\;{\mathrm{time}}_{t}+\beta_{2}\;{\mathrm{sex}}_{s}+\beta_{3}\;\left({\mathrm{time}}_{t}\times {\mathrm{sex}}_{s}\right)+\varepsilon_{t,s}$$

with ${\mathrm{time}}_{t}=t-\mathrm{anchor}$ (centered at 2008), and an AR(1) error structure to account for serial autocorrelation. Parameters were estimated by PROC AUTOREG (SAS 9.4 TS1M9) with Durbin–Watson (DW) diagnostics. We coded sex as an indicator and included an explicit interaction term to allow sex-specific slopes. Because administrative coverage was reported at >100% in some instances, we confined analysis to the open interval (0, 1) by applying a minimal continuity correction to boundary values (0.1% and 99.9%) and truncating rare values > 100% to 99.9%. Primary inferences using Wald tests on linear contrasts included:

⚬ Girls trend: $H_{0}:\beta_{1}=0$ (yearly change among girls).
⚬ Boys trend: $H_{0}:\beta_{1}+\beta_{3}=0$ (yearly change among boys).
⚬ Difference in trends: $H_{0}:\beta_{3}=0$ (boys’ vs. girls’ slopes).

Model fit was assessed via residual plots and information criteria when comparing AR(1) vs. AR(2).

An interrupted-time series (ITS) analysis was conducted to investigate changes in cervical cancer incidence rates (per 100,000) before and after HPV vaccine introduction in 2008 in Panama. Two models were tested, an immediate effect model and a 10-year latency model to account for the time between HPV infection and cervical cancer development (estimated at ~10–15 years). Strong positive autocorrelation was detected; therefore, autocorrelation of up to 2 years was allowed to address the impact of vaccination. Additionally, two time-series regression models were created (immediate and 10-year lagged) using vaccine coverage as the predictor. All models were run in SAS ver. 9.4 TS1M9 (9.4 M9-support.sas.com) and for all comparisons, the significance level was set at α = 0.05.

## 3. Results

The datasets collected contained variables from the HPV vaccine implementation years, the population cancer registry, and varying degrees of pre-implementation data. The main outcomes are described next.

### 3.1. HPV Vaccine Uptake by Doses and Sex over Time

**Figure 1:**
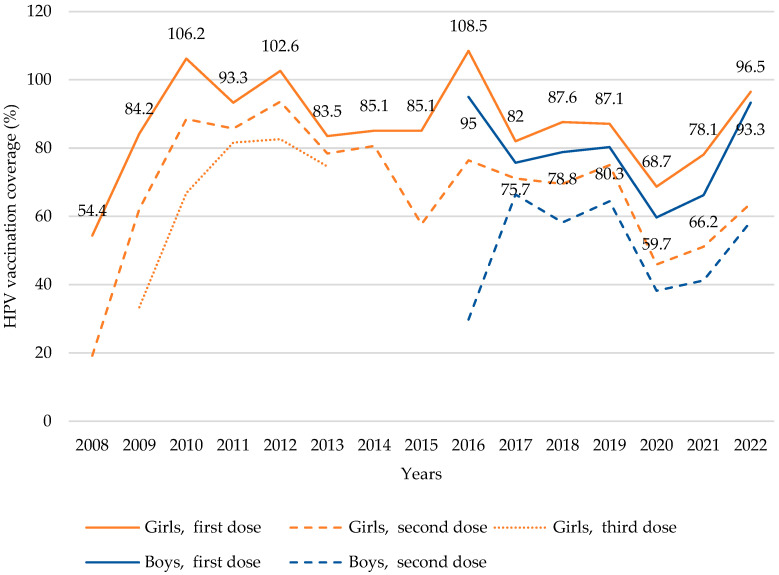
HPV vaccine coverage by dose for girls and boys. Panama, 2008–2022. N.B.1: Coverage numbers correspond to each dose for girls and boys within the schedule, with three doses for girls between 2008 and 2013, two doses for girls between 2014 and 2022, and two doses for boys starting in 2016. N.B.2: Coverage > 100% is discussed in the Data Quality Assurance and Discussion sections.

Since the introduction of the HPV vaccine into Panama’s EPI, a significant increase in vaccine uptake has been observed for the two-dose series, with higher uptake of Dose 1 compared to Dose 2 (Figure 1). Variations in dosage are depicted according to the implementation schedule; for example, the three-dose schedule ran between 2008 and 2013 for girls, with boys included in the program in 2016. Table 1 presents a logit-scale time-series regression analysis depicting vaccine uptake trends that significantly increased over time for Dose 1 (*p* < 0.001) and Dose 2 (*p* = 0.003), with no suggestive difference observed between boys and girls. On the logit scale, girls’ trend between 2008 and 2022 was not significant (*p* = 0.32), nor was the boys’ trend, assessed via the (*β*_1_ + *β*_3_) test, between 2016 and 2022 (p = 0.87). The boys–girls slope difference tested was not statistically significant (*p* = 0.89). The p-value for *β*_1_ + *β*_2_ is not presented in the table.

### 3.2. HPV Vaccine Coverage by Region

**Figure 2:**
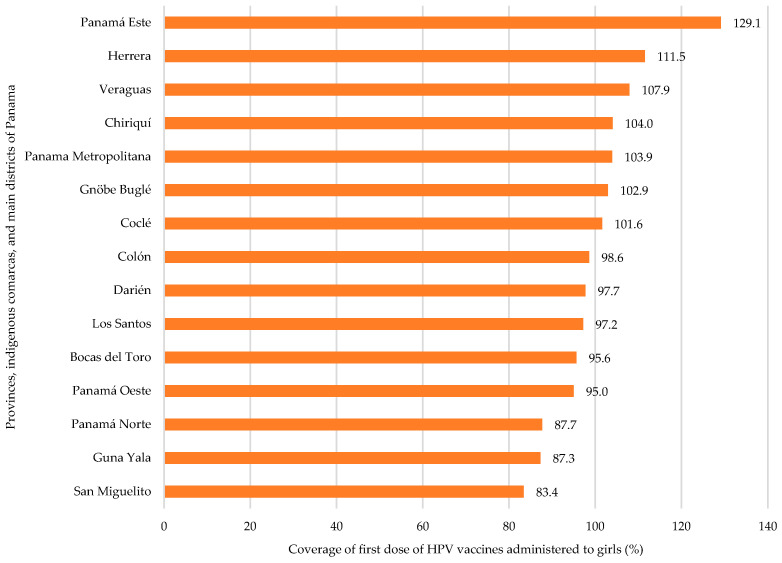
First-dose HPV vaccine coverage among girls by region. Panama, 2015–2022. N.B.: Coverage > 100% is discussed in the Data Quality Assurance and Discussion sections.

Figure 2 depicts the HPV vaccine coverage for Dose 1 among girls by geographic region since the start of the vaccination efforts in 2008. The residential peri-urban region of Panamá Este has the highest coverage rate (129.1%), while peri-urban Panamá Norte (87.6%), the indigenous Comarca of Guna Yala (87.3%), and the densely populated inner-city district of San Miguelito (83.4%) present the lowest coverage rates, below the expected 90%. The rest of the provinces, urban or rural, are closer to 100% coverage, with seven provinces reporting coverage above 100%.

### 3.3. Cervical Cancer Screening

**Figure 3:**
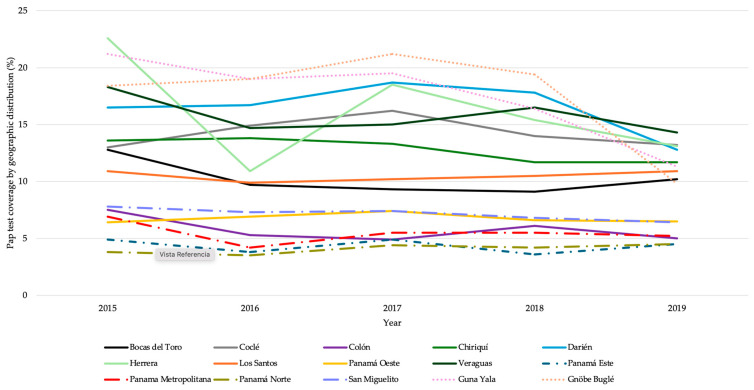
Pap tests by administrative region. Panama, 2015–2019.

Pap tests conducted between 2015 and 2019 indicate low performance of screening procedures (Figure 3), with stark differences observed among regions. In any given year, on average, among all regions and at a national level, Pap tests are performed in 8–10% of the eligible population. A trend regional analysis, through a Chi-square test of independence, indicates steady progression with a significant decrease in rural Herrera in 2016 and the indigenous Ngäbe-Buglé comarca in 2019 (*p* < 0.0001).

### 3.4. Cervical Cancer Incidence over Time

**Figure 4:**
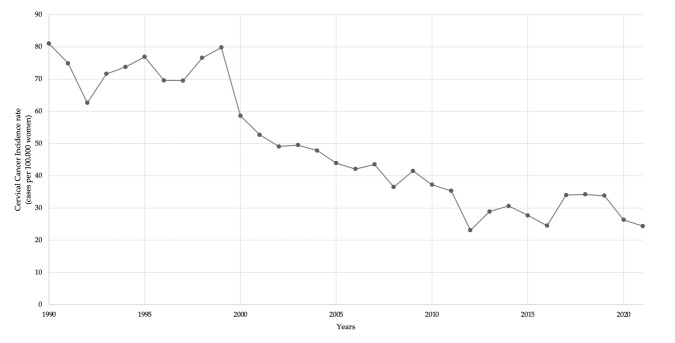
Cervical cancer cumulative incidence rate per 100,000 women. Panama, 1990–2023. The arrow identifies the year of the introduction of the HPV vaccine in Panama’s EPI.

**Figure 5:**
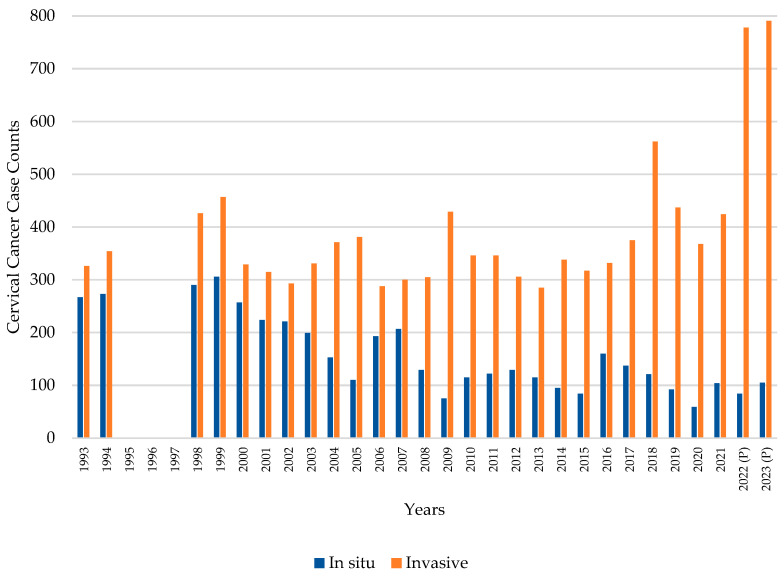
Comparison of in situ and invasive CC diagnoses by year. Panama, 1993–2023. Data missing for 1995–1997.

A downward trend in CC cumulative incidence rate has been observed in Panama since 1990 (Figure 4), in contrast to the increasing trend of invasive CC over time compared to in situ diagnoses presented in Figure 5. This observation has remained steady since 1993, even with a gap in data between 1995 and 1997. Of the total 15,336 reported CC cases between 1993 and 2023, 4426 (28.9%) were classified as in situ, while 10,910 (71.1%) were classified as invasive.

**Figure 6:**
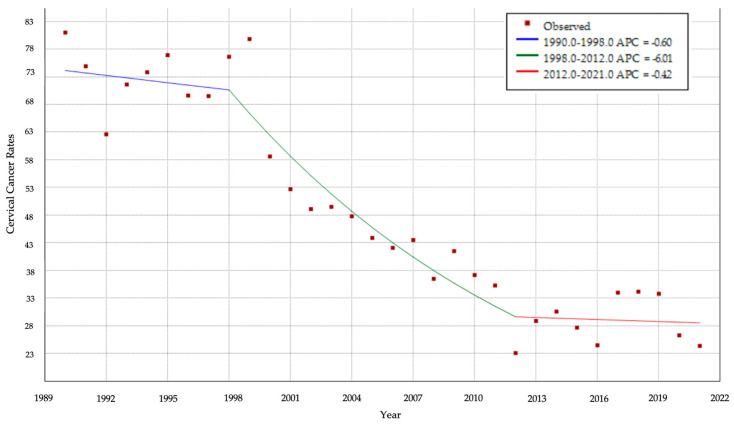
Trend analysis of cervical cancer cumulative incidence rates using Joinpoint. Panama, 1990–2021.

A Joinpoint analysis was conducted for CC cumulative incidence rates in Panama over 32 years (Figure 6). Two change points were identified: in 1998 and 2012. From 1990 to 1998, rates declined slightly (APC = −0.60%/year; blue segment). Between 1998 and 2012, the decline accelerated markedly (APC = −6.01/year; green segment), with cumulative incidence rates falling drastically. This observed decline between 1998 and 2012, is significant. From 2012 to 2021, the trend flattened with a minimal additional decrease (APC = −0.20%/year; red segment).

### 3.5. HPV Vaccination and Cervical Cancer Outcomes

#### 3.5.1. CC Cumulative Incidence over Time

A 10-year latency model shows that the cervical cancer cumulative incidence rate (per 100,000 women) decreased significantly by 2.1 annually in the years prior to 2008. In the year of vaccine implementation, an uptick of 8.4 in the 10-year model estimate is observed, with a statistically insignificant increase of 0.4 per year in the post-implementation period (Table 2).

#### 3.5.2. Cervical Cancer (CC) Mortality

**Figure 7:**
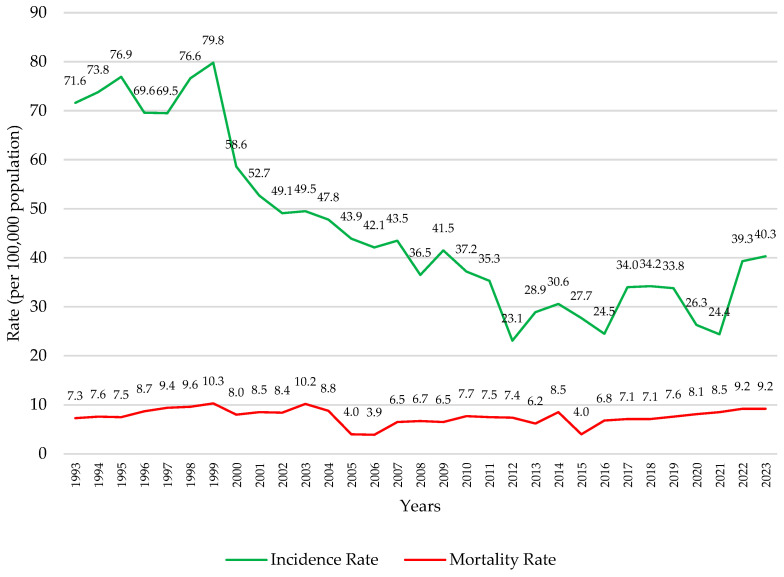
Incidence and mortality rates (per 100,000 women) of cervical cancer over time. Panama, 1993–2023 *. * Years 2022 and 2023 depict preliminary data.

**Figure 8:**
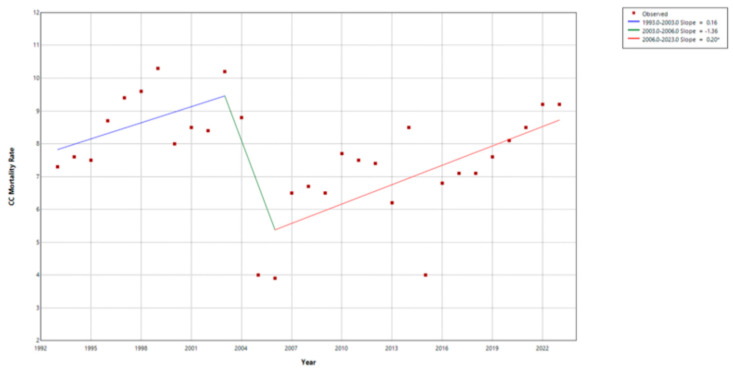
Trend analysis of cervical cancer mortality rates using Joinpoint in Panama, 1993–2023.

**Figure 9:**
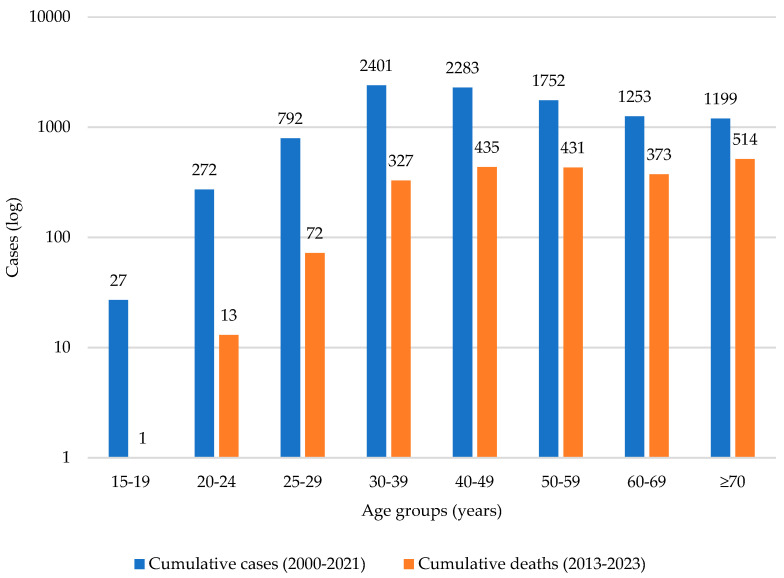
Comparison of CC cumulative cases and cumulative deaths by age groups, Panama, 2000–2023.

CC mortality rates have been analyzed since 1993, with a steady increase since 2006, as compared to incidence rates (Figure 7). A Joinpoint analysis identifies two change points in CC mortality rates, one in 2003 and one in 2006. From 1991 to 2003, CC mortality slightly increased (APC = 0.16%/year; blue segment), and decreased sharply between 2003 and 2006 (APC = −1.36%/year; green segment). From 2006 to 2023, CC mortality significantly increased (APC = 0.20%/year; red se46gment) (Figure 8). Figure 9 shows the cumulative CC cases between 2000 and 2021 and compares them to the cumulative number of deaths attributed to CC between 2013 and 2023 by age group. Increased diagnosis and death are observed among age groups between 30 and 69 years.

## 4. Discussion

This analysis triangulates and assesses national data on HPV vaccination coverage, screening practices, and cervical cancer incidence and mortality in Panama, to understand historical tendencies to date and establish a comprehensive foundation for future impact evaluations and research studies. The analysis aims to identify trends and gaps in prevention efforts and to serve as a reference point for future research on HPV-associated cancers.

This is an important initial step, as Panama was the first country to introduce the HPV vaccine as part of its EPI. Although a complete causal link between the HPV vaccine and observed outcomes cannot be demonstrated in these analyses, the initial observations serve as a reference point for future necessary clinical, epidemiological, and behavioral studies once additional immunization and cancer data are generated.

### 4.1. HPV Vaccine Uptake

The HPV vaccine uptake is encouraging in Panama for both girls and boys (Figure 1 and Table 1). The Panamanian government’s goal of reaching the PAHO/WHO promoted initiative of implementing appropriate milestones for cervical cancer elimination by 2030 is underway, through the 90:70:90 targets [31,32,33]. Nonetheless, considerable work is still pending, with a growing need to evaluate the impact of the HPV vaccination program [42]. In the 1980s, Panama participated in landmark studies that increased understanding of HPV transmission and related cervical cancers, including the male factor in transmission [27]. Hence, the current analysis includes the vaccination uptake of boys, as an important factor in the transmission of HPV [28,29].

Between 2008 and 2020, the 90% minimum national vaccine coverage increased overall, with some sharp concavities. Some of the coverage decreases tend to be related to governmental changes in administration and lapses in local or foreign funding, especially during the SARS-CoV-2 (COVID-19) pandemic. Seven out of fifteen (47%) geographic regions reported HPV vaccine coverage rates in excess of 100% (Figure 2). The results of this observational analysis indicate that the inclusion of the HPV vaccine within Panama’s EPI is associated with overall adequate coverage in vaccination of the intended population, through a traditional strong culture of vaccination [43,44,45] (Figure 1 and Figure 2), introduced during the sanitation process involved in the construction of the Panama Canal.

*Vaccine Data Considerations.* Various factors drive coverage above 100% in the processes followed by the EPI. Mass community-level blanket vaccination campaigns occur and are recorded on top of regular vaccinations [30]. Thus, the EPI counts the number of doses given and divides them by the estimated population, a common practice in population registries in LMICs. Furthermore, the latest Panamanian census data was obtained in 2020–2021 amidst the SARS-CoV-2/COVID-19 pandemic, with some concerns in the methodology and results that may lead to over- and underestimates of the population by region [36]. Evidence of this observation resides on the actual record of live births for 2021 (*N* = 74,147) which does not match the census estimate of live births for the same year (*N* = 66,498). Finally, while adjustments are made by the EPI to report high coverages to international organizations, raw internal primary data may yield estimates of over 100%, usually reported in this manner.

Nurses vaccinate children who arrive without immunization cards, resulting in an increased number of new cards, duplicating vaccination efforts. While it is generally true that these additional doses will not account for every unvaccinated individual in a population, they may do so when vaccine coverage is relatively high and after repeated mass vaccination campaigns, which are fairly common in Panama. For example, during the 2023 Vaccination Week of the Americas (VWA), where vaccination efforts take place across borders for populations that lack access to regular health care including indigenous and migrant populations, the EPI of Panama identified 21,779 boys and girls 10–14-years old, of these 6186 (2992-boys and 3194-girls) were vaccinated against HPV, corresponding to 18% of the 33,772 vaccines administered in this age group [30]. This has been the team’s previous finding when working with other vaccines, such as pertussis [39] and polio eradication, where one author serves as an evaluating commission member [46]. Although annual revisions of the data to account for over-immunization are ideal, there is a two-year delay on final reporting. The EPI team is currently working on aligning the EPI database to the INEC census data and updating the reporting system to correct denominator issues for future follow-up and quality assurance. Thus, real-life digital data would warrant a positive intervention for data management in the immunization program [44] and assist in data harmonization efforts to reduce redundant resource expenditure [45,47,48,49,50].

### 4.2. Cervical Cancer

Prior to the introduction of the HPV vaccine, especially at the start of the millennium (Figure 4, Figure 5, Figure 6 and Figure 7), there was a decreasing trend in the incidence rates of CC among Panamanian women [51,52,53,54,55,56,57]. In the 1990s, Panama introduced a proactive approach to developing public health policies for the prevention and early detection of cervical cancer [52,53,54]. Regulations focused on the importance of the Pap test, early detection of precancerous lesions, and the prevention of HPV infection [52,53]. Although the authors were unable to identify peer-reviewed literature or agency reports to corroborate this information, through narrative informal discussions, the authors learned that there were prevention campaigns generated around Pap testing outreach initiatives at primary care settings [58,59]. However, especially noticeable are the elevated cervical cancer mortality rates (Figure 7 and Figure 8) [55,56,57].

Despite the availability of free or low-cost Pap tests in Panama, women’s regular participation in cervical cancer screening remains low—approximately 10% among all eligible women nationally—especially in densely populated areas, such as the district of San Miguelito, where the prevalence of HPV infection exceeds 44% [58]. A lower proportion of Pap tests is observed among women ages 20 to 49 years by the MoH (15% coverage among eligible women), despite this being a priority group for screening [55,56,57].

*Mortality.* Of concern is the significant increase in mortality between 2006 and 2023. Once an irregular Pap test result is observed, the patient is referred to a colposcopy for observation and possible biopsy. Between 2019 and 2022, a total of 21,134 colposcopies were performed; of these, 19,037 (90%) were among younger women 20–59 years of age. Anecdotal discussions with local oncologists indicate that younger women are approaching the oncology institute with advanced stages of cervical cancer, warranting unsuccessful treatment outcomes. In addition, low Pap test uptake is observed (8–10%). In sum, this is also a topic for future in-depth research, through structural and sociocultural factors which might play a role in cervical cancer outcomes. Once more, this present analysis is observational, and it serves as a reference point for future research studies that include more in-depth analysis of the current situation of HPV, cervical, and other forms of HPV-related cancers in Panama.

Decades of Pap test–based screening to detect pre-cancerous cervical lesions in certain LAC countries have not had a major impact in reducing CC incidence and mortality rates [14,15]. The increased mortality due to CC, albeit systematic screening through Pap tests, is observed in other countries in LAC, and consistent with the existing literature, can be explained within the regional context [14,15].

Further observations on diagnosis and mortality (Figure 8 and Figure 9) denote aspects related to system navigation and follow-up. Once a woman is screened, Pap results might take a prolonged period to be reported, and CC diagnosis tends to be late, delaying access to treatment. Invasive CC behavior is noticeably more frequent than in situ CC in Panama; however, in the existing population data sources, stages of cancer are not identified. The time series analysis (Table 2) allows us to conclude that, to date, the introduction of the HPV vaccine has not significantly changed cervical cancer incidence [60,61,62,63]. The authors suspect that this finding can be attributed to the premature and slow changes in cervical cancer incidence due to HPV immunization, which are just beginning to become apparent. A longer period of vaccine impact analysis and effectiveness within the local context is recommended at this point.

Although a direct epidemiological association cannot be established based on the existing data, the results serve as a reference point for future studies, including modeling and the effectiveness of the vaccine. The hypothesis is that higher HPV vaccination coverage in the population leads to lower incidence rates of CC over time; at this moment it is too early to confirm this has occurred in Panama [64,65,66,67,68,69]. Not all HPV infections result in the development of cervical lesions and may be eliminated by the immune system in a short period of time [70]. The extent to which viral infections are cleared remains a major unresolved question and needs to be explored in other types of cancers (oropharyngeal, anal, penile, vulvar) and respiratory afflictions, and among key at-risk populations [70]. Participation studies and including the HPV vaccine as a preventative measure showcases Panama’s early prioritization of this public health issue. Other countries are implementing self-testing and HPV-based screening in place of Pap tests, with promising cytological accuracy outcomes [71].

### 4.3. Health Education and Promotion

To contextualize health education efforts, we conducted a comprehensive examination of peer-reviewed literature, government documents, and official reports from PAHO, WHO, and CDC, supplemented by manual searches of Panamanian archives. No formal documentation of systematic prevention campaigns was identified. Nonetheless, it was apparent that information was disseminated through mass media, particularly television, featuring spokespersons, such as physicians and public health professionals [72]; additionally, close female social networks—mothers, friends, coworkers, neighbors—and male partners, also influence the decision to get tested [46,58,59]. At the time of this publication, the MoH was working on the development of cervical cancer prevention and control guidelines. It is unclear what the current knowledge of HPV vaccine and related cancers is in Panama; future studies, such as the THRIVE-25 study conducted in Australia, provide a pathway for this type of inquiry [73]. Previous knowledge measures based on age, educational level, and income showed that more educated women were more likely to be exposed to vaccine information [46,58,59,74].

### 4.4. Sociocultural Considerations

Previous studies indicate that Panamanian women present misconceptions regarding HPV, and that they believe the Pap test detects or cures vaginal infections or serves as a pregnancy test [46,58,59,74]. Although most have heard about the HPV virus and its sexual transmission, less than half are aware of its association with cervical cancer, other forms of cancer, or genital warts, while others mistakenly believe HPV can be cured with antibiotics or that its symptoms are linked to the menstrual cycle [46,58,59,74]. Only one-third of women surveyed in Panama correctly identified that the Pap test is used to detect cervical cancer [74]. Cultural and psychosocial factors such as fear, shame, and mistrust of male health professionals also negatively influence participation. Added to this are logistical barriers such as domestic responsibilities, lack of time, childcare, and transportation difficulties, which limit access to health services. Traditionally, Panamanians support a culture of vaccination, where vaccine hesitancy is quite rare. Regarding the HPV vaccine, it was introduced through the school programs and was mandatory at age 10, before the onset of sexual debut. Any social stigma of the HPV vaccine is ameliorated in Panama with a cervical cancer prevention focus.

### 4.5. Structural Considerations

An overall increased trend in vaccination was established throughout 2008–2021, with an observed decrease in vaccination rates to 42% in 2020, attributed to the SARS-CoV-2/COVID-19 pandemic. The school system and all primary healthcare facilities in Panama closed for 81 weeks following strict lockdowns [60], affecting vaccine access. Panama experienced a 51.5% drop in HPV doses administered in the first half of 2020 compared to the same period in 2019 during the SARS-CoV-2/COVID-19 pandemic. The decrease in coverage during this period could mean that cohorts of girls and boys were less protected through immunization. This phenomenon could translate into a future increase in cases of persistent HPV infections with possible development of related cancers.

Populations in LMICs often face a variety of barriers to accessing health care, such as lack of transportation, time, low awareness of prevention methods, low awareness of risk, and low socioeconomic levels [14,15]. Many of these barriers persist in Panama and prevent populations from accessing the HPV vaccine and seeking cervical cancer care, projecting a complex interaction between structural barriers, misinformation, and sociocultural norms that hinder effective cervical cancer prevention in Panama [46,58,59,74].

Geographic distribution affects vulnerable populations, such as indigenous groups, where hard-to-reach areas face limited access to immunization through the EPI program. To address these barriers, each year the MoH, in coordination with PAHO, conducts the VWA. The annual blanket immunization campaign aims to reach vulnerable populations facing difficulties accessing health services due to poverty or geographically remote locations [30]. The campaign intends to increase coverage of key vaccines (e.g., COVID, HPV, rotavirus, hepatitis B, influenza, polio, measles, and pertussis), to address misinformation at the community level, and to respond to any inquiries the population might present [30,46,58,59,74]. In this campaign, 34% of the intended population was vaccinated against HPV [30]. The EPI offers immunization at the population level to all populations, including those that would be considered vulnerable, such as rural communities and indigenous Comarcas, as depicted in Figure 1 and Figure 2. Community outreach and blanket immunization activities are common in Panama.

Despite the reduction in cervical cancer incidence and the successful implementation of the HPV vaccine in the EPI, Panama still has one of the highest cervical cancer rates [55,56,57,60,61]. The pandemic also affected cervical cancer screening and educational campaigns, as most public health interventions focused on COVID-19 prevention and immunization, and the clinics remained closed for screening services. What makes the CC outcomes remarkable is that younger women who are affected by invasive CC are diagnosed at later stages. Continued monitoring and follow-up are recommended as recent developments in herd immunity are observed, with up to 71.6% protection against the most carcinogenic HPV types [66,68]. Further studies in this regard are necessary to address CC in Panama.

To continue reducing the cervical cancer rates in Panama, there needs to be increased outreach efforts, HPV and cervical cancer education, prevention methods based on formative research, follow-up of patients, and the creation of data harmonization techniques for real-time analysis. Social and cultural factors must be considered when designing health education on HPV and related cancers, improving the effectiveness of future prevention methods, and increasing health literacy among the intended population [46,58,59,73,74]. Improved resources are necessary to escalate awareness campaigns and access to screening methods, which are vital for reducing cervical cancer rates [46,58,59,72,73,74]. As the Panamanian government enacts the new Science Against Cancer research initiative [75], it is expected that short- and long-term cancer outcomes in Panama will be studied and addressed, and, consequently, positively affect public policy with adequate political resolve and resources.

### 4.6. Limitations

The present analysis is observational, and its intent is not to establish causality. The findings of this exploration rely on the accuracy of the data collected by the MoH through the EPI, the National Cancer Registry, Epidemiological Bulletins, and INEC. This study may be affected by selection bias as it focuses on measuring cumulative incidence rates and, therefore, cannot account for the entirety of the population at risk. In all cases, these cumulative incidence rates are an approximation since the denominator includes the subgroup in its entirety. There is reduced generalizability of this analysis as it primarily focuses on Panama. However, it could be applied to countries or regions with similar contexts [14,15].

## 5. Conclusions

The present study provides a baseline for future original experimental research in epidemiological, clinical, and behavioral sciences, including additional modeling of projected CC and other HPV-related cancer incidence, low- and high-grade lesions, total of averted cases of HPV-related diseases, deaths, and the economic impact of the vaccine. Throughout this extensive analysis, we have identified high HPV vaccine coverage and a steady decrease in cervical cancer incidence, albeit an increase in mortality—particularly among younger women—highlighting critical gaps in prevention efforts. This analysis also provides context to a country that pioneered the implementation of the HPV vaccine, where screening remains low (approximately 10% of the intended population) and mortality is high, with deaths reported among younger age groups. A further analysis of mortality is recommended through future clinical studies.

These findings underscore the need for integrated strategies that strengthen data systems, improve early detection, and address structural and sociocultural barriers. By serving as a reference point for future research and policy development, this work supports Panama’s progress toward achieving the 90:70:90 targets and informs the design of studies that can evaluate vaccine impact and optimize cervical cancer control. There is a tangible need in Panama to automate data collection and reporting, conduct data harmonization and quality assessments, and create access to immunization and public health outcomes, providing open access to researchers. Real-world studies conducted elsewhere, with linkage of immunization data and screening databases, allow for direct monitoring of virological, histological, and cancer outcomes [45,47,48,66,67,68,69]. The use of data sources which are not designed to systematically collect, organize, and report figures to support research also underscores a need to develop and implement a robust methodology for the data system that provides insight into vaccine effects and consequences of different health policy decisions [45,47,48,76,77]. Providing these data harmonization possibilities in Panama would allow for future studies of HPV-related cancers—such as cervical, penile, oropharyngeal, and anal—which are currently not studied in this country, especially with at-risk, immunosuppressed populations [78]. Structural barriers to immunization and screening should be a strong focus of future government initiatives. The current analysis, the first of its kind in Panama, serves as a reference point for future studies to be conducted, especially as we move toward the elimination of HPV and related cancers as measures of vaccine impact.

## Figures and Tables

**Table 1:** HPV Dose 1 uptake: logit-scale time-series regression 2008–2022.

Term	Estimate	Standard Error	*p* Value
$\mathrm{Intercept}\;(\beta_{0})$	9.52	3.11	0.01
$Time\;(\beta_{1})$	−0.44	0.43	0.32
$Sex\;(\beta_{2})$	−5.81	16.03	0.72
Time $*\;\mathrm{Sex}\;(\beta_{3})$	0.20	1.49	0.89

MSE = 56.58; DFE = 17; *R*^2^ = 0.2; AR = 1; DW = 1.98 (*p* = 0.39). Abbreviations: AR, autoregressive; DFE, degrees of freedom for error; DW, Durbin–Watson statistic; MSE, mean standard error.

**Table 2:** Interrupted time series analysis of cumulative CC rates in Panama, 1990–2021.

**Interrupted Time Series Analysis**		
	**Immediate Model Estimate** **(MSE = 32.20, rDF = 28, ** ***n*** ** = 32)**	**10-Year Model Estimate** **(MSE = 36.65, rDF = 28, ** ***n*** ** = 32)**
Intercept	81.30 (*p* < 0.001)	80.12 (*p* < 0.001)
Time before intervention	−2.18 (*p* < 0.001)	−2.10 (*p* < 0.001)
Year of intervention	−8.86 (*p* = 0.12)	8.37 (*p* = 0.33)
Time after intervention	1.59 (*p* = 0.03)	0.44 (*p* = 0.89)
Durbin–Watson Statistics	2.03 (*p* = 0.34)	1.83 (*p* = 0.18)
**Time Series Regression with Vaccine Coverage**		
	**Immediate Model Estimate** **(MSE = 27.74, rDF = 12, ** ***n*** ** = 14)**	**10-Year Model Estimate** **(MSE = 7.43, rDF = 2, ** ***n*** ** = 4)**
Intercept	38.73 (*p* = 0.001)	42.45 (*p* = 0.047)
Vaccination Rates	−0.09 (*p* = 0.39)	−0.15 (*p* = 0.30)
Durbin–Watson Statistics	1.75 (*p* = 0.35)	1.73 (*p* = 0.24)

Abbreviations: MSE, mean standard error; rDF, residual degrees of freedom.

## Data Availability

No new data were created for this analysis. Public data supporting results can be found, at the Ministry of Health of Panama, Epidemiological Bulletins (https://www.minsa.gob.pa/contenido/boletin-estadistico-ano-2021final-2022preliminar-2023preliminar [accessed on 16 November 2025]), the National Cancer Registry of Panama (https://www.minsa.gob.pa/contenido/registro-nacional-del-cancer [accessed on 16 November 2025]), Cervical Cancer Prevention (https://www.minsa.gob.pa/sites/default/files/programas/normas_de_prevencion_cacu.pdf [accessed on 16 November 2025]), and the National Institute for Statistics and Censuses (https://www.inec.gob.pa/publicaciones/Default3.aspx?ID_PUBLICACION=491&ID_CATEGORIA=3&ID_SUBCATEGORIA=10 [accessed on 16 November 2025]). Additional public data supporting results can be found at Ministry of Health of Panama Statistics section (https://www.minsa.gob.pa/informacion-salud/estadisticas-de-salud [accessed on 16 November 2025]), which includes epidemiological data, demographics, prevention, the population-based cancer registry, and the international classification of diseases.

## Abbreviations

The following abbreviations are used in this manuscript:

APC	annual percentage change
AR	autoregressive
CC	cervical cancer
CDC	Centers for Disease Control and Prevention
DFE	degrees of freedom for error
DW	Durbin–Watson statistic
EPI	Essential Program on Immunization
HPV	human papillomavirus
INEC	National Institute for Statistics and Census
ITS	Interrupted time series analysis
JRF	Joint Reporting Form on Immunizations
LAC	Latin America and the Caribbean
LMIC	low- and middle-income countries
MoH	Ministry of Health of Panama
MSE	mean standard error
PAHO	Pan-American Health Organization
Pap	Papanicolaou
rDF	residual degrees of freedom
SRH	sexual and reproductive health
STIs	sexually transmitted infections
VWA	Vaccination Week of the Americas
WHO	World Health Organization
